# Supplementary figures and images for: Clinical and laboratory features of COVID-19 illness and outcomes in immunocompromised individuals during the first pandemic wave in Sydney, Australia
Source: PLoS One. 2023 Nov 1;18(11):e0289907. doi: 10.1371/journal.pone.0289907 (PMC10619805; doi:10.1371/journal.pone.0289907)

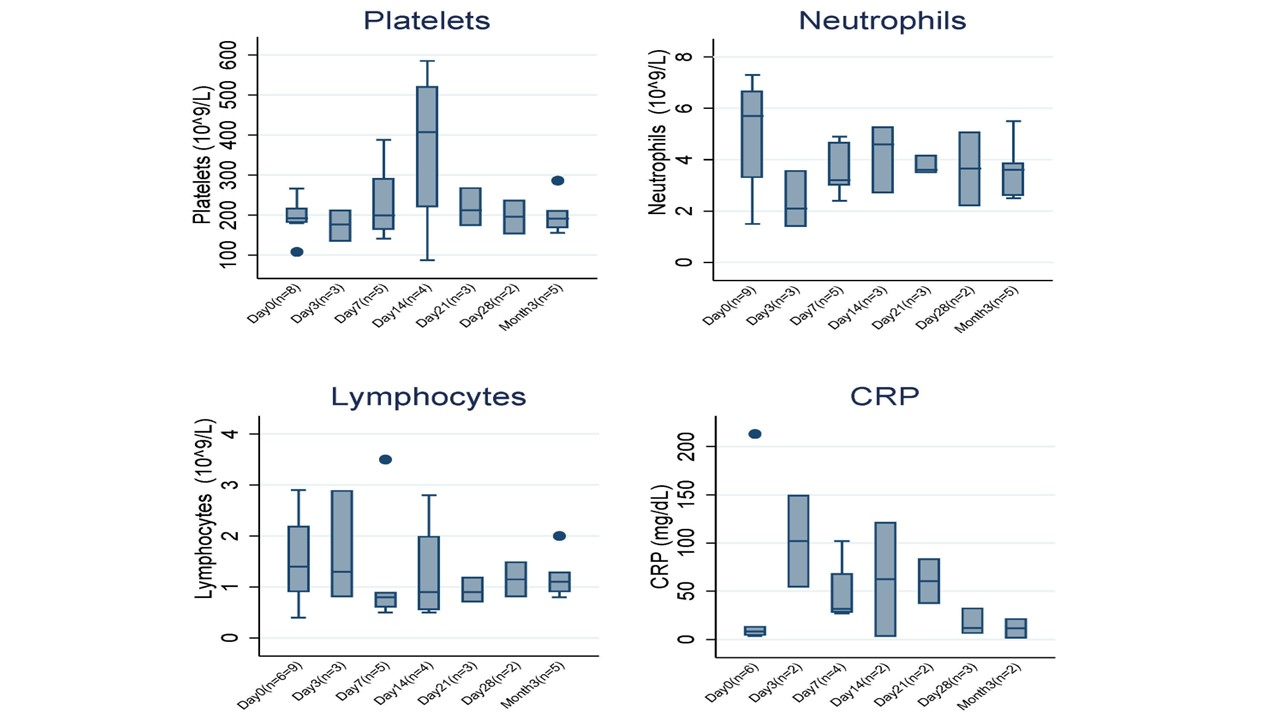

Supplement: S1 Fig — (TIF) [file pone.0289907.s001.tif]
