# Supplementary material for: Clinical and laboratory features of COVID-19 illness and outcomes in immunocompromised individuals during the first pandemic wave in Sydney, Australia
Source: PLoS One. 2023 Nov 1;18(11):e0289907. doi: 10.1371/journal.pone.0289907 (PMC10619805; doi:10.1371/journal.pone.0289907)
Supplement: S1 Table — (DOCX) [file pone.0289907.s002.docx]

**Supplemental Table 1.** **Medication list** (data available for 19 participants)

| **Participant** | **Primary diagnostic criteria** | **Underlying immunosuppressive condition** | **Medications** |
| --- | --- | --- | --- |
| 1 | Haematologic/oncologic conditions | Cutaneous melanoma | Anti-PD-1, Anti-PDL1 |
| 2 | Haematologic/oncologic conditions | Cutaneous melanoma | Anti-PD-1, Anti-CTLA-4 |
| 3 | Haematologic/oncologic conditions | Cutaneous melanoma | Anti-PD-1, Anti-CTLA-4 |
| 4 | Haematologic/oncologic conditions | Follicular lymphoma | Rituximab, Intravenous immunoglobulin (IvIg) |
| 5 | Haematologic/oncologic conditions | Refractory myeloma | Methylprednisolone |
| 6 | Haematologic/oncologic conditions | Malignant neoplasm of other connective and soft tissue | Anti-PD-1 |
| 7 | Haematologic/oncologic conditions | Malignant immunoproliferative diseases | Ruxolitinib |
| 8 | Haematologic/oncologic conditions | Chronic transfusion dependent myelofibrosis | Ruxolitinib |
| 9 | Haematologic/oncologic conditions | Malignant neoplasm of oesophagus | Trastuzumab (Herceptin), Capecitabine |
| 10 | Primary/acquired immunodeficiency | HIV infection | Elvitegravir/cobicistat/emtricitabine/tenofovir alafenamide |
| 11 | Primary/acquired immunodeficiency | HIV infection | Dolutegravir+darunivir/cobicistat |
| 12 | Primary/acquired immunodeficiency | HIV infection | Dolutegravir+darunivir/cobicistat |
| 13 | Primary/acquired immunodeficiency | HIV infection | Not on HIV treatment |
| 14 | Primary/acquired immunodeficiency | Unknown | Mycophenolate Mofetil, Prednisone |
| 15 | Primary/acquired immunodeficiency | Autoinflammatory Disorders | Methotrexate |
| 16 | Primary/acquired immunodeficiency | Autoinflammatory Disorders | Etanercept, Prednisone |
| 17 | Secondary immunosuppression | Psoriasis | Secukinumab |
| 18 | Secondary immunosuppression | Multiple sclerosis | Fingolimod |
| 19 | Secondary immunosuppression | Rheumatoid arthritis | Methotrexate, Sulfasalazine |
| 20 | Secondary immunosuppression | MOG Antibody Demyelinating Syndrome | Tecifidera,  Ocrelizumab |
