# Supplementary material for: Clinical and laboratory features of COVID-19 illness and outcomes in immunocompromised individuals during the first pandemic wave in Sydney, Australia
Source: PLoS One. 2023 Nov 1;18(11):e0289907. doi: 10.1371/journal.pone.0289907 (PMC10619805; doi:10.1371/journal.pone.0289907)
Supplement: S2 Table — *Only positive PCR results are reported. (DOCX) [file pone.0289907.s003.docx]

**Supplemental Table 2.** Number of participants with positive COVID-19 PCR positive results over time, by primary immunocompromising diagnostic category*

|  | Primary diagnostic category | | | Total  N=9 |
| --- | --- | --- | --- | --- |
|  | **Haematologic/oncologic conditions**  **N=5** | **Secondary Immunosuppressive/immunomodulatory treatment**  **N=3** | **Primary/Acquired Immunodeficiency**  **N=1** |  |
| Last time point with positive PCR test |  |  |  |  |
| Day 3 | 1 | 2 | - | 3 |
| Day 7 | 1 | - | - | 1 |
| Day 14 | - | - | - | - |
| Day 21 | 1 | 1 | - | 2 |
| Day 28 | 1 | - | - | 1 |
| Month 3 | 1 | - | 1 | 2 |

*Only positive PCR results are reported.
