# Supplementary material for: Clinical and laboratory features of COVID-19 illness and outcomes in immunocompromised individuals during the first pandemic wave in Sydney, Australia
Source: PLoS One. 2023 Nov 1;18(11):e0289907. doi: 10.1371/journal.pone.0289907 (PMC10619805; doi:10.1371/journal.pone.0289907)
Supplement: S3 Table — (DOCX) [file pone.0289907.s004.docx]

**Supplemental Table 3**. Clinical outcomes of participants at three months after enrolment.

|  | **Hospitalisation** | | **Invasive mechanical ventilation** | | **Death** | |
| --- | --- | --- | --- | --- | --- | --- |
|  | **Month 3**  **n (%)** | **Time from enrollment to outcome (median, IQR), days** | **Month 3**  **n (%)** | **Time from enrollment to outcome (median, IQR), days** | **Month 3**  **n (%)** | **Time from enrollment to outcome (median, IQR), days** |
| **Haematologic/oncologic conditions** | 3 (25%) | 3 (1-7) | 1 (8%) | 14 | 1 (8%) | 90 |
| **Secondary Immunosuppressi**on | 3 (38%) | 7 (1-28) | 2 (25%) | 3 (3-3) | 1 (13%) | 90 |
| **Primary/acquired immunosuppression** | 3 (43%) | 3 (3-3) | 0 (0%) | NA | 0 (0%) | NA |
| Total | 9 (33%) | 3 (1-5) | 3 (11%) | 3 (3-14) | 2 (4%) | 90 (90-90) |
